# “I Am I and My Bacterial Circumstances”: Linking Gut Microbiome, Neurodevelopment, and Depression

**DOI:** 10.3389/fpsyt.2017.00153

**Published:** 2017-08-22

**Authors:** Juan M. Lima-Ojeda, Rainer Rupprecht, Thomas C. Baghai

**Affiliations:** ^1^Department of Psychiatry and Psychotherapy, University of Regensburg, Regensburg, Germany

**Keywords:** gut microbiome, neurodevelopment, depression, neuroimmune–endocrine system, microbiome–gut–brain axis, hypothalamic–pituitary–adrenal axis, energy homeostasis, nutrition

## Abstract

Recently, there has been renewed interest in the role played by microbiome in both human health and human disease. A correct equilibrium between the human host and their microorganisms is important for an appropriate physiological function. Extensive research has shown that microbes that inhabit the gastrointestinal tract—or gut microbiota—are involved not only in both nutritive and digestive activities but also in immunological processes. Moreover, the gut microbiome influences both central nervous system and energy homeostasis. An altered gut microbiome has been associated with the pathophysiology of different diseases, including neuropsychiatric disorders. Apparently, both environmental—diet, exposition to antibiotics, and infections—and host-genetic factors have a strong influence on gut microbiome, modulating the risk for neuropsychiatric illness. Also, early life disruption of the microbiome–gut–brain (MGB) axis has been associated with an increased risk of developing depression later in life, suggesting a link between gut microbiome, neurodevelopment, and depression. This review aims to contribute to this growing area of research by exploring the role played by the gut microbiome in neurodevelopment and in the etiology of the depressive syndrome, including nutritional, immunological, and energy homeostasis approaches.

The title of this review is a paraphrase of the maxim from Ortega y Gasset: *Yo soy yo y mi circunstancia, y si no la salvo a ella no me salvo yo* (“I am I and my circumstance; and, if I do not save it, I do not save myself”) (1).

## Introduction

Our world has an extraordinary diversity of life forms. A key aspect of them is their individuality; each creature on Earth—unicellular or multicellular—has its own characteristics. So, on our planet, it is possible to identify, in different ecosystems (Table 1), numerous animals, bacteria, plants, fungi, and archaea. Each creature plays an important and unique role in its habitat. On Earth, there are several ecosystems and all of them are characterized by complex interactions between different organisms.

**Table 1:** Brief glossary.

Glossary
**Anaerobic bacteria**: microorganisms that have the characteristic that they are able to growth without oxygen
**Anorexigenic cells**: cells associated with the loss of appetite
*Binary fission*: in bacteria, it is an asexual reproduction where the bacterium duplicates its deoxyribonucleic acid, generating a new daughter cell—or more than one daughter cell—with the same characteristics that the mother or original bacterium
**Dysbiosis**: term employed to refer to important disruptions at the indigenous bacterial level, including both structural and compositional alterations
**Ecosystem**: a complex system where living creatures interact with their environment and its physical components, being all its constituent interconnected elements
**Germ-free (GF) mice**: GF mice are animals without microorganisms’ colonization in or on their bodies
**Gnotobiotic**: it refers to an environment where all its microorganisms are controlled or known
**Gut-brain axis**: a complex network characterized for a strong connection between the gastrointestinal tract and the central nervous system (CNS). It is a bidirectional system
**Immunosenescence**: age-related chronic and progressive process at the immune level that is associated with an increased pro-inflammatory activity
**Inflamm-aging**: an increased inflammatory response associated with the development
**Lactic acid bacteria**: microorganisms that produce lactic acid
**Meconium**: earliest feces from infants
**Microbiome**: term employed to refer to the combination of microorganisms and their genetic materials. Sometimes this term is used in the scientific literature as synonym of microbiota
**Microbiome-gut-brain (MGB) axis**: bidirectional connection between the gastrointestinal tract and the CNS, including the gastrointestinal microbiome. Frequently this term is used as synonym of microbiota–gut–brain axis
**Microbiota**: microorganisms that inhabit a particular environment, e.g., a specific anatomical region
**Microbiota-gut-brain (MGB) axis**: a bidirectional axis that communicates the intestinal microbiota with the brain
**Minor physical anomalies**: congenital physical changes associated with insults during the development
**Orexigenic cells**: cells that are associated with the induction of appetite
**Polyphenols**: compounds of chemical kind that are produced by plants
**Prebiotics**: kind of dietary fibers that are beneficial for both microbiota development and function
**Probiotics**: term used to refer to living microorganisms which ingestion has been associated with host’s benefits at the health level
**Xenobiotics**: foreign substances that are founded in the environment and that are usually synthetic chemicals

Microorganisms such as bacteria are important for the human life. Some products are obtained because of fermentation (2), including bread, beer, wine, cheese, and yogurt (2). In general, bacteria have influence not only in our eating culture (3) but also in different sociocultural and economical aspects of human life (3). Microbiome (Table 1) in and on human bodies is a dominant feature of our individuality (3), increasing the number of publications about microbiome–human bodies interactions (3). In general, host–microbiota (Table 1) ecosystem is a story of cooperation (4, 5), reflecting past and present of both host and their microorganisms (4, 5). In order to avoid any confusion, in this paper, we will use the term “microbiome” to refer to both the microbes and their genetic materials, as unit, and not as synonym of the term “microbiota.” Also, for the purpose of this manuscript, we will focus on gastrointestinal bacterial microbiome.

There are differences in the microbiome composition among vertebrates (6). Also, individuals from the same species show some variations in their microbiome (4, 6). Each anatomical region has its microbiome (6), which has its own characteristics (6). The gastrointestinal tract in humans is a sanctuary of living organisms, the most of our microbiota inhabit this region (7). It has been pointed out that if we are born sterile, bacteria that inhabit our gastrointestinal tract may come from the external environment—outside the boundaries of our bodies—(4).

Gastrointestinal microorganisms proliferate by binary fission (Table 1) (4). This ecosystem is a dynamic one (4), where changes at the level of the microbiota composition have been associated with physiological dysfunction and health alterations (8, 9). Recently, there has been renewed interest in the role played by microbiome in the neuropsychiatric sphere (10). It has been noted that gastrointestinal bacteria have influence on behavioral and cognitive processes (11). Moreover, there is a growing body of literature that recognizes the importance of gut microbiome in neurodevelopment (12).

If we want to understand our individuality, we should accept the importance of the innumerable creatures that are around us and to understand the interactions between the different individuals that are part of the numerous ecosystems on Earth. In general, the world that surrounds us is important to define our individuality. This situation is better expressed by the famous maxim of the illustrious philosophe José Ortega y Gasset, a well-known intellectual who wrote at the beginning of the twentieth century: *Yo soy yo y mi circunstancia, y si no la salvo a ella no me salvo yo* (“I am I and my circumstance; and, if I do not save it, I do not save myself”) (1). The main goal of this review is to explore the relationship between gut microbiome, neurodevelopment, and depression. Also nutritional, immunological and energy homeostasis approaches with relevance in the neurobiology of the depressive syndrome are explored. We conducted an English language literature search using the biomedical database PubMed. To improve the quality of our manuscript, we included complementary information.

## Microbes in Human Health and in Human Disease: A Story of Co-Evolution

For centuries, humans and bacteria have evolved together (8). We have a strong interaction with our indigenous microbiota (8, 13). From a gnotobiotic (Table 1) condition during the intrauterine life to a complex microbiome–human interaction with a begin at birth (8), bacteria in and on human bodies are influenced by both environmental and host-genetic factors (8). The constitution of microorganisms that inhabit our organism is defined by host-genetic material (9). Evidence suggests that microbiome is able to mediate host gene expression (11). Surprisingly, most of the cells in and on human body are microorganisms or microbes (14). Also, microorganisms in humans have complex genomes. For example, the gastrointestinal microbiome has a genome that is around 100 times more numerous than human’s one (15), being the products of a large number of these microorganism genes significantly important for the physiology of the host (16). Using deoxyribonucleic acid (DNA) extraction from a European human fecal sample collection with 124 individuals it has been characterized the human gut microbiome (17), which has 3.3 million genes (17), being the most of them from bacteria—99.1% (17). Interestingly, bacteria have the ability to obtain genes that they need to continue to live (4). Since microorganisms that cohabit within the same environment are able to swap genes (4), the dominant bacterial population gain control over processes of swapping genetic material (4). Also, inheritance of the microbiota has been suggested (6), particularly this one associated with the mother (6), which is a stable vertical inheritance (4). It has been proposed that if we wish to have a better knowledge about ourselves, we should look at our genome, including our indigenous bacterial genome (13). In general, humans and their indigenous microbiota have co-evolved together, establishing a dynamic ecosystem.

There is evidence about interactions between animal hosts and their microbiome (6, 9, 18–20). Environmental factors, such as diet (6, 9, 18, 19), infections (6, 9), and antibiotics (6, 9, 18), may alter these interactions (6, 9, 18, 19). Early in development, the acquisition of bacteria in body structures such as the gastrointestinal tract is important to develop an adequate tolerance to antigens (13). Moreover, it has been suggested that indigenous microbiome is important for an appropriate host development during extrauterine life (13). The growth of microorganisms in the host’s gastrointestinal tract depends also of both gut physical—intestinal movements (19)—and chemical—both digestive enzymes and acid secretions (19), oxygen quantity (21), pH (potential of hydrogen) level (21)—factors (19, 21). It has been proposed that our daily intake modulates gut microbiome (19, 22), being carbohydrates an important source of nutrients for gut bacteria (9, 18). Since gut microorganisms require nutrients for their development (19) and the bacteria’s host is the most important supplier of nutrients for them (19), then host’s feelings such as satiety and hunger are important factors defining gut bacterial composition (19). In general, host–microbiota interaction is an important factor modulating bacterial energy homeostasis at the gut level (19). A hypothesis from the second half of the twentieth century pointed out that the composition of the indigenous organisms that inhabit the gastrointestinal tract is limited by the effective management of nutrients between species of bacteria (23). More recently, it has been noted that the inclusion of vegetables, whole grains, fish, and fruits in our diet is positive for our gastrointestinal microbiome (22). Intake of omega-3 fatty acids, folic acid, S-adenosyl-methionine, l-tryptophan, vitamin B_12_, and vitamin D has been associated with a mental health improvement (22). Interestingly, dietary interventions, including supplements of minerals, essential fatty acids (EFAs) and vitamins, have been associated with a better behavior in humans (24). For example, results from a double blind, placebo-controlled, randomized study with adult prisoners showed that a nutritional supplementation of EFAs, minerals, and vitamins was associated with an antisocial behavior improvement (24).

One of the most significant findings to emerge from different studies about human microbiome is that perturbations of the host–microbiota are associated with an increased risk for different diseases (6, 10, 25), including gastrointestinal diseases (6, 25), dermatological illness (6), obesity (6, 25), type 1 diabetes (25), neurodegenerative illness (26), neuropsychiatric diseases (10), neuroimmune diseases (26, 27), and rheumatic disorders (6, 25), where changes in the interactions between microbiome and host immune system have been proposed as important etiological factors (6). Sometimes our immune system is unsuccessful in restricting our indigenous microbiota (8, 13), then the microorganisms are not more positive at all. Microbes are considered pathogens when they are able to produce a disease (8, 13). Despite the importance of microbiota–human host cooperation, there remains a paucity of knowledge on microbes–human host equilibrium. The Nash equilibrium—a concept from the game theory (8)—has been used to try to explain “the rule” of this cooperation (8). According to this concept, the players—or both indigenous bacteria and human body—know the rules of the game—or equilibrium—(8). When one of the players departs from the established rules, this player will be sanctioned—maybe by host immunity—, taking a weak position (8). However, biological interactions, such as this one observed between microorganisms and human body, are “more dynamic and complex games” to be clarified under this theory.

The microorganisms that inhabit the gastrointestinal tract, including their genome are known as gut microbiome (25). A significant number of microorganisms—10 trillion (9) to 100 trillion (9, 15–17)—is part of the adult gut (15). The most of them are anaerobic bacteria (Table 1)—99.9% (11, 16, 20)—and inhabit the distal part of the gastrointestinal tract (15). The gastrointestinal tract is entirely inhabited by microorganisms (21), in ascendant quantity from the duodenum to the last part of the colon (21). There is an important presence of cytophaga–flavobacterium–bacteroides (9, 15, 16, 18, 28) and Firmicutes (9, 15, 16, 18, 28). Other examples of dominant habitants of the gastrointestinal tract are: *Streptococcus* in the distal esophagus and *Helicobacter* in the gastric region (18). In general, the gut flora has subjects from *Firmicutes, Bacteroidetes, Verrucomicrobia, Actinobacteria, Proteobacteria*, and *Fusobacteria* phyla (29), being the most of the intestinal microorganisms part of the first two phyla (29). Normally, the gut offers a stable environment to these bacteria (11). These microorganisms are able to communicate with each other (13, 15) and they have a strong connection with the host’s organism (15). Gut microorganisms are dynamic inhabitants of the gastrointestinal tract (19), processes of growing, lysis, and elimination are part of the mechanisms that stabilize the bacterial population (19). In general, gut microbiome is beneficial for the human host (11, 18, 25); these microorganisms are involved with digestive and nutritive processes (11, 18, 20, 25). For example, gut bacteria are related to the metabolism of lipids, proteins, carbohydrates, and vitamins such as B and K (18). Interestingly, there is evidence that gastrointestinal microorganisms play a crucial role in metabolizing medicaments and xenobiotic substances (Table 1) (18). Also, the gut microbiome reinforces host immunity (11, 18, 20, 25) and influences the formation of gut vasculature (20). In the intestine, gut microbiome modulates both adaptive and innate immunity (18, 25). For an adequate gastrointestinal epithelium, the gut microbiome is important (27). At the gastrointestinal level, bacteria are involved in the presence of a “physiological inflammation” (11), which is important for a gut in good health (11). However, gut microbiome dysbiosis (Table 1)—or important changes at the level of the structure and the composition of the microorganisms (9)—has been linked with pathological autoimmune processes in the nervous system (27).

It is important to note that it has been suggested that the first 36 months of extrauterine life are fundamental defining host–bacterial composition (28) and that the sequence of the bacterial transition during the first months of life is difficult to define (30). Environmental factors, including vaginal delivery and breast milk (6, 28), influence strongly early exposition to bacterial communities in humans (6, 28), especially to lactic acid bacteria (Table 1) (6). Also, environmental factors are able to alter the gut microbiome equilibrium (7, 28, 31). Some other environmental factors that have been reported as elements that could influence gut microbiome characteristics are factors, such as prebiotics and probiotics (Table 1) consumption, family composition, cultural practices, locality, antibiotics, and mother–infant interactions (30). Together, these data indicate that microbiome–human interaction is a story of diversity and adaptability (15). However, the whole association between microbiome and different diseases is still unknown (6).

### The Gut Microbiome and the Nervous System: The Microbiome–Gut–Brain Axis

**Figure 1:**
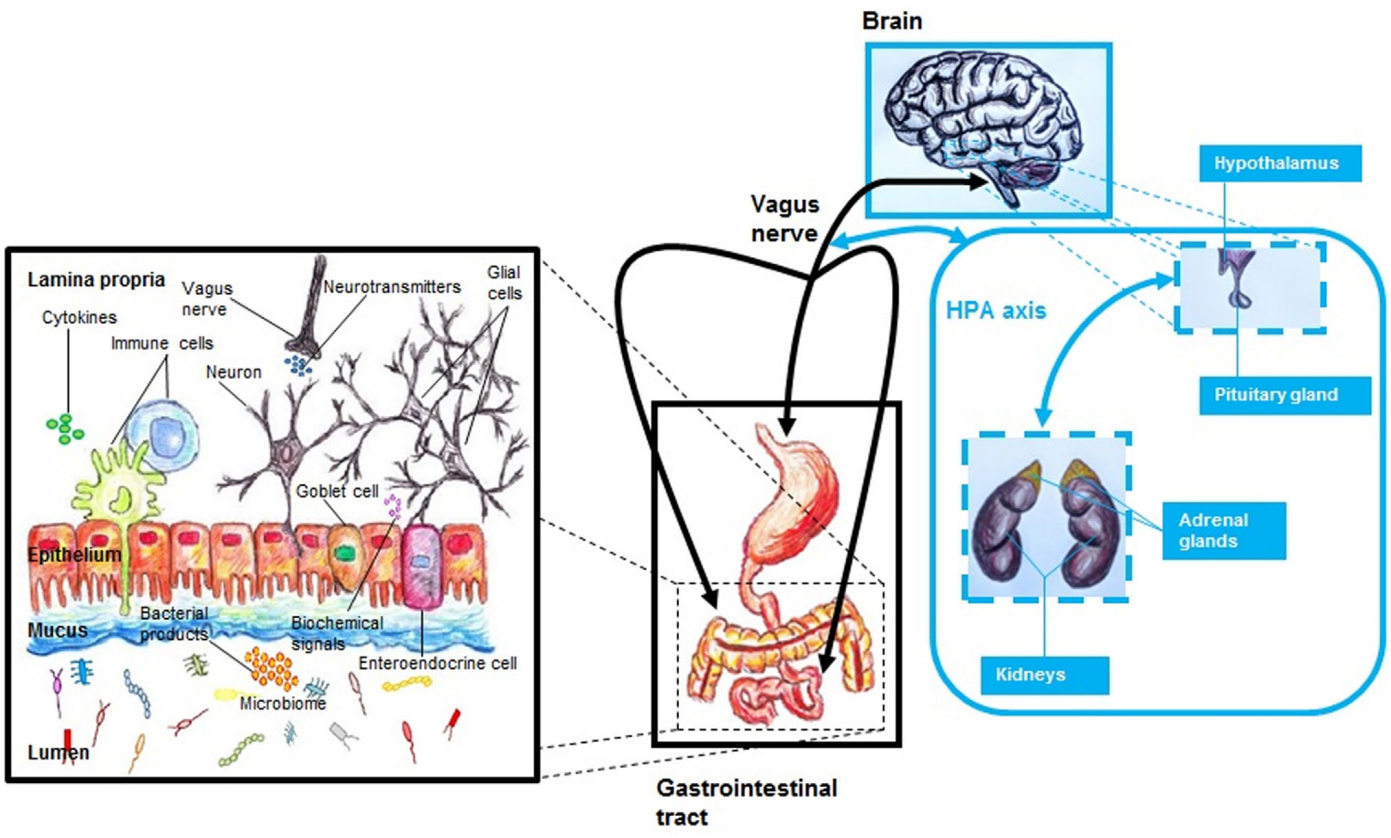
The MGB axis, including the hypothalamic–pituitary–adrenal (HPA) axis. The MGB axis is a bidirectional system that links the gastrointestinal tract with the brain. It is a complex system characterized by a neuroendocrine–immune communication. The gut microbiome influences the function of the brain by modulation of both immune and endocrine systems, HPA axis, neurotransmitter pathways, and growth factors. Alterations of this network—that includes numerous molecules and cells—may be the basis of pathological processes.

The gut–brain (GB) axis (Table 1) is a complex system that integrates the gut with the nervous system (10, 16). A main characteristic of this system is its bidirectionality (10, 11, 16, 26, 31), where a neuro-endocrino-immunological connection is implicated (16) (Figure 1). In general, the GB axis is important regulating gastric and intestinal function and energy homeostasis (11). The tenth cranial nerve or vagus nerve, with its both afferent and efferent fibers (32), is a link between the gastrointestinal tract and the brain (32, 33). The anterior vagal trunk bifurcates into three limbs: hepatic, anterior or ventral gastric, and celiac branches (34). In the gastrointestinal tract, at the gastric level, in the lamina propria or mucosa lamina propria, in both external muscularis propria, and in the myenteric plexus are neuronal terminals (34). In the gastric mucosa, afferent fibers of the vagus nerve are able to identify the presence of ghrelin and leptin (33). Also, at the level of the small intestine, it has been noted a complex net of vagal afferent fibers (34), which has similarities to these observed at the gastric level (34). It has been suggested that afferent fibers of the vagus nerve could identify the presence of luminal contents (33, 34), being this possible by the presence of mechanosensors (e.g., serosal receptors, mucosal touch receptors, muscular tension receptors) (33, 34). Apparently, chemoreceptors of the vagal afferent fibers are reactive to different molecules, such as glucagon-like peptide-1 (GLP-1) (33, 34), interleukin-1β (IL-1β) (34), serotonin (5-HT) (33, 34), somatostatin or growth hormone inhibiting hormone (34), and cholecystokinin (CCK) (33, 34), being duodenal vagal afferent fibers reactive to both 5-HT and CCK (33), and at the level of the ileum, colon, and rectum, afferent fibers of the vagus are reactive to peptide YY and GLP-1 (33).

The nucleus tractus solitarius (NTS) of the dorsal vagal complex of the brainstem integrates nervous signals from the gastrointestinal tract (34, 35). Also, these vagal and spinal signals are later transmitted to the hypothalamus (35). Seemingly, the activation of nerve cells from the NTS associated with the afferent vagal system is mainly dependent on *N*-methyl-d-aspartate receptors (35), but this is not clear (34). Also both the infundibular or arcuate nucleus (ARC)—the third largest hypothalamic aggregation of nerve cells (36)—and the paraventricular nucleus (PVN) in the hypothalamus receive projections from the NTS (35). There is evidence that signals from the afferent vagal system are also transmitted to brain regions outside the hypothalamus, such as insula, stria terminalis, and amygdalae (34).

Since the gut microbiome may influence the central nervous system (CNS) (10, 31), it has been proposed to include the microbiome as part of this axis (10), being known as microbiome–gut–brain (MGB) axis (10) or microbiota–gut–brain axis (Table 1) (16). Gut microbiome is able to mediate host gene expression (11). This is particularly important if we consider that this could include genes related to CNS function (11). It has been suggested that gut microbiome influences the CNS by modulation of the hypothalamic–pituitary–adrenal (HPA) axis, immune system, neurotransmitter pathways, and growth factors (Figure 1) (10). A link between disrupted gastrointestinal microbiome and both behavioral and cognitive alterations has been noted (10, 37). Also, an adequate performance at the learning and memory level has been related to a functional microbiome (37). Changes at the level of the HPA axis have been associated with microbiome composition (16), including both behavioral and cognitive alterations that are apparently linked with molecules, such as norepinephrine (NE), 5-HT, brain-derived neurotrophic factor (BDNF), and dopamine (DA) (16). Apparently, gut microbiome is able to modulate the function of limbic system (37), including regions such as amygdala and hypothalamus (37). Emotional stress appears to impact gut microbiome composition (11, 16, 31). Also, the pathophysiology of neuropsychiatric disorders, such as depression (10, 37), schizophrenia (10), anxiety (37), and autism (37), appears to involve gut microbiome changes (10, 37). Interestingly, behavior appears to play a role to define the characteristics of gastrointestinal microbiome (37). Individual level of social interaction has been linked directly with microbiome composition (37), including behavioral characteristics on the list of modulation factors for gut microorganisms (37). In this bidirectional system, the gastrointestinal microbiota seems to modulate both neuronal function and behavior (38), including behaviors such as anxiety and appetite behavior (38). Apparently, these processes involve the interaction of both the MGB and HPA axis (38). Also, appetite regulation could be modulated—*via* a endocrino-neuronal vagal-hypothalamic communication (19)—by molecules that are produced by gut microorganisms (19). It has been noted that brain reactions *via* stress are able to alter gastrointestinal function (16), including gut microbiome composition changes (16), and early life disruption of the MGB axis have been associated with later life neuropsychiatric disorders such as depression (10).

To date, numerous studies using probiotics have attempted to demonstrate MGB axis’ communication (16). Administration of probiotics has been associated with neurophysiological changes (16), where it seems to include the participation of different molecules, such as 5-HT, BDNF, DA, NE, and corticotropin releasing factor (16). Between the different species that are employed as probiotics to improve gastrointestinal equilibrium *Bifidobacterium* and *Lactobacillus* are well-known (38). Bacterial reconstitution of *B. infantis* has been associated with an improved HPA axis’ response to stress in a neurodevelopmental animal model (39). Also, probiotic administration of *Lactobacillum rhamnosus*, for a month (40), is enough to modulate both neuronal gene expression and behavior in animals (40). In general, this information tries to expose the importance of the MGB axis at the neurobiological level (39, 40).

### The Microbiome–Gut–Brain Axis and the Immune System

The immune system is an important part of the MGB axis. The immune system, including both adaptive and innate immune systems (27), mediates the communication between bacteria, gastrointestinal tract, and CNS (27). Also, a gastrointestinal microbiome is of great significance for an adequate gut immunity (41). Host’s microbiota is the main source of microorganism antigens for the host (20). In humans, during development host’s microbiota is an important element modulating the immune system maturation (20), being host’s intestinal microorganisms involved in the formation of the lymphoid tissue at the gut level (20).

It is important to remember that the intestine is an anatomical barrier that makes possible the separation between visceral systems and indigenous bacteria (42). Most of the cells that are part of the intestinal epithelium are columnar epithelial cells (41). Proteins related to the innate immune system, such as toll-like receptor (TLR) 1–3 (TLR1-TLR3), TLR5, TLR9, and nucleotide oligomerization domain 2—which are part of the pattern recognition receptors (41)—, and chemotactic factors are expressed by epithelial cells (41). In the small intestine, T-lymphocytes—which are known as intraepithelial lymphocytes (41)—are part of the epithelium (41). Interestingly, gut microorganisms are able to modulate epithelial gene expression *via* molecules related to the immune system such as TLR (20). Peyer’s patches are well-known lymphoid structures that are part of the small intestine (41), being important gut-associated immune structures (41). The mucus produced by goblet cells in the intestine, in both large and small portions (41), integrates a functional barrier that has an important role as defensive layer against pathogens (9, 41). Both the glycoprotein mucin and different polysaccharides are the main components of this mucus (9), which is degraded principally by gut microbiota (9). The large intestine is a B-lymphocytes-rich zone (41). Dysbiosis at the gastrointestinal level has been associated with an elevated activity of molecules associated to the immune system such as cytokines (42). As consequence of this elevated activity, it has been recognized an intestinal barrier dysfunction (42), which is accompanied for chronic inflammation (42). Gut immunoglobulin (Ig) A is an important regulator of microbiome composition (41). Epithelial cells are provided with antimicrobial molecules that together with IgA improve both transport and anti-bacterial protection at the intestinal level (43). The intestinal epithelial barrier—which is a barrier against pathological microorganisms (44)—reduces the risk of intestinal inflammation (44). Mucosal enteric glial cells (EGCs) are important part of the immune-homeostasis of the gut microenvironment (44). EGCs are immune cells that are able to present antigens to T-lymphocytes (44). It is known that these cells are implicated in the production of different factors—such as neurotrophins, glial-derived s-nitrosoglutathione, glial-derived neurotrophic factor, transforming growth factor-β1, and 15-deoxy-Δ^12,14^-prostaglandin J2 (15dPGJ2) (44)—associated with the homeostasis of the neuroimmune system (44). Moreover, neurotransmitter signaling alterations of the serotonergic system in both nervous system and gastrointestinal tract have been associated with intestinal inflammatory disorders (45), suggesting a link trough 5-HT between gut and brain (45).

Different microorganisms that are part of the gut microbiome have been linked with anti-inflammatory or inflammatory immune reactions (41). *Bacteroides fragilis* and *Lactobacillus* have been associated with anti-inflammatory responses (41). Also, protective intestinal inflammation has been linked with gut microorganisms such as segmented filamentous bacteria (41). A positive inflammatory modulation has been related to bacteria such as *Clostridia* (41). Apparently, indigenous microbiota has an immunomodulatory potential in gut mucosa (41), regulating both immune cells and molecules (41). Interestingly, it has been observed that there is a link between chronic inflammation and gut microbiota (21), where high level of intestinal oxygen alters normal bacterial composition and induces inflammation (21). Apparently, high level of gut oxygen drastically damages gut-microbiota equilibrium (21), reducing anaerobic population and increasing aerobic inhabitants (21). In general, the MGB axis has a strong link to the immune system.

### The Microbiome–Gut–Brain Axis and Energy Homeostasis

The gastrointestinal tract is an important organ that has different functions, such as nutritional, absorptive, and homeostatic. The small intestine has numerous enzymes that mediate nutritional and digestive processes (21), being in the intestine the small segment where the most of the absorptive and digestive activities take place (21). Reabsorption of water, degradation of dietary fiber, and vitamins’ uptake occur mainly in the large intestine (21). The gastrointestinal tract has its own microbiome. Gut microorganisms are important for both homeostasis and carbohydrate metabolism in human host (9). Around 10% of host’s caloric intake has a bacterial origin (20). Gastrointestinal microbiome can be directly influenced by our habitual food (46), being diet a complex environmental factor that plays an important role shaping gut microbiota (46). Apparently, host’s feeding behavior rhythms have an impact in the gut bacterial distribution (19), being host’s circadian rhythm alterations able to trigger a change in the quantity of intestinal microorganism that normally predominates during the diurnal period (19). Complex molecules such as polysaccharides are metabolized for bacteria that inhabit the gastrointestinal tract (9), working altogether as a metabolic organ in the human body (9). Moreover, gut microbiota makes these polymeric carbohydrate molecules an energy source for the host (9). Intestinal bacteria are involved in the metabolism of macronutrients and the metabolites produced for these microorganisms are able to modulate epigenetic changes in the host (46). A diet, including a high amount of fiber, polyphenols (Table 1)—present in green tea, nuts, and fruits (46)—and omega-3 fatty acids—available in fish oil (46)—has been linked with bacterial changes—e.g., a rise in the number of both *Lactobacillus* and *Bifidobacteria* (46)—at the gastrointestinal level (46). Gut microbiota—including microorganism, such as *Clostridium coccoides*, Actinobacteria, *Roseburia, Bifidobacteria, Lactobacillus, Escherichia coli, Peptostreptococcus, B. thetaiotaomicron*, and *Enterococcus faecalis* (46)—is associated with the metabolism of these dietary components (46). Interestingly, alterations at the level of the gut bacterial population’s growth have been linked with the presence of a disrupted metabolic control (19). It has been suggested that the intestinal microorganisms could regulate their own homeostatic control of energy balance by using their host’s energy homeostasis mechanisms (19). An increment or reduction in the number of gut bacteria could induce a feeling of lack of food or satiety in the host (19).

The CNS has a pivotal role in the homeostatic regulation of energy balance (35, 47). Different chemical and neuronal signals from peripheral organs are involved in the energy homeostasis process (32, 35, 47). In the brain, the hypothalamus plays an important role regulating body metabolism (32, 35, 47). This brain region is involved in the control of body weight, food intake, and thermoregulation (47). Both peripheral hormones, such as insulin and leptin, and numerous nutrients modulate—*via* hypothalamus—the control of energy homeostasis (48). Leptin and insulin are implicated in the CNS in the food reward system (48). Also, there are pieces of evidence about the expression of receptors for leptin and insulin in diverse brain areas (48), including a complex system in the CNS for energy regulation (48). The initial contact with consumed food takes place in the gastrointestinal tract (35), being the GB axis fundamental to preserve—as product of neuroendocrine communication (35)—the balance between energy intake and energy expenditure in the body (35). Apparently, the homeostasis of gut microorganisms plays an important role in the energy homeostasis of the host (19), being appetite control a fundamental mechanism for host–bacteria homeostasis (19). The integration of this microbiota–host communications appears to be *via* gastrointestinal tract-hypothalamus (19).

In general, neuronal population from the ARC and the PVN is important regulating energy homeostasis (47). Both orexigenic and anorexigenic cells (Table 1) are present in the ARC (47). Agouti-related protein (AgRP) and neuropeptide Y (NPY) are expressed for the first kind of neurons (47), while anorexigenic nerve cells express proopiomelanocortin (POMC) (47). In the brain, orexigenic and anorexigenic neurons have projections to both hypothalamic—lateral hypothalamus, PVN, ventromedial nucleus, and dorsomedial hypothalamic nucleus—and extra-hypothalamic areas (32). An increased need for food has been associated with AgRP—by melanocortin 4 receptor (MC4R) related action—and NPY—by NPY receptor linked activity—(32). Both POMC and NPY neurons are known as main sites of expression of leptin receptors (49). Leptin, which body concentration is depended of adipose tissue grade (50), is a hormone with implications in both body weight and metabolism (50). Leptin has a satiety factor function through inhibition of both AgRP and NPY cells and activation of POMC neurons (32). In the case of POMC cells, it has been proposed that the effect of leptin on this kind of neurons occurs in the ARC (49, 50), where the frequency of action potentials is increased by leptin (49). As mechanism for this action, it has been proposed that leptin induces both depolarization of POMC nerve cells and decrement of gamma-aminobutyric acid release onto POMC cells (49). Also, the peptide hormone known as α-melanocyte stimulating hormone (α-MSH), an agonist of the MC4R (32), is produced by POMC neurons from the ARC (47). The NTS and the vagal system are involved increasing appetite by ghrelin action (32). Ghrelin activates orexigenic neurons and suppresses anorexigenic cells (32). In general, gut microbiota play an important role in human energy homeostasis.

## The Gut Microbiome and Neurodevelopment

Our bacterial composition, this one that we have during our adult life, starts to be acquired during our early life (42), being the mother the main element defining the characteristics of the infant’s microbiome (30). Normally, the gut is firstly exposed to microbes at birth (7). Apparently, human’s first bacteria come from mother’s vagina and excrement (4). There is some evidence to suggest that the placenta has a microbiome (51, 52). Data from metagenomic information indicate the presence of an indigenous microbiota in human placenta (51, 52). Since bacteria—such as *E. coli, Staphylococcus epidermidis* and *E. faecalis* (53)—have been isolated from meconium (Table 1) from healthy newborns (53), it is believed that there is an intrauterine contact between healthy fetus and microorganisms (53). Also, it is believed that there is a fetus–maternal–bacteria interaction (52), since it has been noted a bacterial presence in the amniotic fluid, from mothers, and meconium, from neonates, both without antibiotic contact (52). However, with little scientific experiences, very few data available and discussible methodologies, caution must be applied, and the question about a possible prenatal human microbiome is still open. In general, the physiological gut composition is not clear (30), but it is known that the gastrointestinal bacterial composition is different during childhood and adulthood (30). It is important to note that *E. coli* is one of the first microorganisms that inhabit the infant gut (30).

A link between microbiome composition and neurodevelopment has been proposed (16, 38), where functional changes in different brain regions (e.g., hypothalamus, amygdala, hippocampus, and striatum) are present (16). Microorganisms could influence neurodevelopment (16, 31). Development of the enteric nervous system (ENS)—which have EGCs as main component (44)—is regulated by gastrointestinal microorganisms (38). EGCs are important transmitting signals related to the ENS (44). Gut microbiome composition has been associated with neuronal connectivity development (16). Also, bacteria seem to be associated with the quality of neuronal circuitries during the postnatal life (28). It has been discussed that alterations of the gut microbiome, during the prenatal and the postnatal period (10), may affect the normal neurodevelopment (10). It has been hypothesized that neurodevelopment could be mediated by host–microorganisms *via* MGB axis (42). Since molecules involved in the neural development such as neurotrophic factors, including BDNF (54), have been related with the MGB and HPA axis (16), and they are susceptible of modulation *via* epigenetic regulation (54), microbiome alterations could influence strongly on the neurodevelopment. This epigenetic regulation may be result of gut microbiota-host chemical communication (55), where bacterial bioactive metabolites—e.g., butyrate (55)— and signaling molecules—e.g., peptides and endotoxins (55)—make possible this communication (55). Moreover, brain development may be modulated by the MGB axis involving the participation of the neuro-endocrino-immunological system (43).

Recently, an association between a disrupted microbiome and abnormal neurodevelopment has been noted (56). Germ-free (GF) mice (Table 1)—which are animals with a microbiota deficit from birth (56), being a helpful tool to study microbiome–brain interactions (56)—have showed neuroanatomical changes in brain areas, such as amygdala and hippocampus, during the late adolescent/early adulthood period (56). These changes included neuronal morphological aberrations, such as basilar dendrites with thin spines (56), hypertrophy of aspiny interneurons from the basolateral complex (BLA) (56), pyramidal neurons with long dendrites (56), short pyramidal neurons in the ventral hippocampus (56), and reduction of stubby spines of the hippocampal pyramidal neurons (56). Also, it has been proposed that gut bacteria have an important impact in early neurodevelopment (42), with the participation of both microglia and endocrine molecules as modulator factor (42) and emotional stress and/or antibiotics—for example—as risk factors (42). Published in 2004, results from experiments from a developmental model using GF mice have reported that alterations at the level of early postnatal microbiota affect both the HPA axis and the limbic system (39), including both elevated level of plasma adrenocorticotropic hormone and corticosterone after stress exposition and reduced level of BDNF in brain regions, such as hippocampus and cortex (39). All these data where observed when GF mice were compared with mice having a specific microbiota (39), suggesting an important neurodevelopmental role for indigenous microbiota regulating HPA axis (39). In general, in the human life cycle, there are prenatal and postnatal environmental factors that play an important role defining the characteristics of each microbiome (6, 31), and during the different host life cycles have been observed microbiota age-related changes that could be determined by microbiota’s genes (6).

Evidence suggests that there is an important role played by gut microbiome in human brain development. However, the whole association between the MGB and human neurodevelopment is still unknown.

## The Gut Microbiome and the Depressive Syndrome, from a Neurodevelopmental Perspective

Human intestine formation is concluded around the 12th week of intrauterine life (57). Early during the prenatal period begins the formation of the MGB axis (57, 58). Main cells of the ENS are product of the migration of neural crest cells (57). Neural crest cells can be observed in the early gastrointestinal organ around 7–12 weeks of gestation (57). It is important to note that the interstitial cells of Cajal have a different origin (57), being of mesenchymal basis (57). It has been reported that neurons such as 5-HT enteric cells are born early during the intrauterine life (58). In general, 5-HT is an important neurotransmitter in the MGB axis (45). This molecule is susceptible of modulation *via* gut bacteria (45). Together with 5-HT, molecules such as TLRs and short chain fatty acids have been proposed as participants in the GB axis’ function that show a strong link to gut microbiota composition (43). In the newborn, during the first weeks of extrauterine life (58), there is a strong functional connection between both enteric neuronal cells and glial cells (58), being a critical period for alterations induced by environmental factors (58). Expression and activation of the receptor tyrosine kinase RET seem fundamental for ENS development (58). During the early development the immune system and its molecules—e.g., cytokines (58)—and cells—e.g., macrophages (58)—play an important role modulating the formation of neuronal circuits at the gastrointestinal level (58). During the neonatal period, a positive modulation of the immune system has been associated with breastfeeding (59). It seems that the basis of the MGB axis and its link with the neurodevelopment is by modulation of cells and molecules that integrate the neuroendocrine-immunological system (43).

It has been suggested that the presence of a neuropsychiatric disorder is frequently associated with the existence of a gastrointestinal illness (16, 31). Also, gastrointestinal diseases have been linked with gut microbiome dysbiosis (60). Early life stress such as neonatal maternal separation has been connected with intestinal dysfunction (61). A characteristic gut microbiota in depression has been reported in humans (62, 63), including both high level of Bacteroidales and low Lachnospiraceae composition in a first study (62), and both an increased *Alistipes* population and Enterobacteriaceae level and low grade of *Faecalibacterium* in a second one (63) in depression (62, 63). Interestingly, it has been noted that *Faecalibacterium* has a protective effect for neuropsychiatric disorders such as depression (63). In general, this information suggests a relation between the gastrointestinal system and the nervous system, with a strong communication at the level of the MGB axis.

The development of our MGB axis begins early in life, including a complex neuroendocrine-immunological modulation. It seems that stress factors, such as emotional stress and gut microbiota alterations, play an important role modulating the risk for diseases such as depression.

### The Neurodevelopmental Theory of Depression: An Overview

Neurodevelopment is an important process to understand the depressive syndrome. It is a dynamic process with a long trajectory (64), from gestation to adulthood (64). The neurodevelopmental theory of the depressive syndrome has been reviewed recently elsewhere (65). Each human brain region has its maturation time period (65), being the most relevant morphological modifications during the prenatal period, childhood, and early adolescence (65). The beginning of the human neurodevelopment is around the third and fourth week of intrauterine life (66, 67) with the neurulation as first event (66–68). It has been reported that the dorsolateral prefrontal cortex is one of the final brain regions to mature (69).

It has been noted that the depressive syndrome and both structural and functional abnormalities at the level of the limbic system, which are associated with the disease, are product of early neurodevelopmental alterations (65). Risk factors, such as altered fetal period (70), maternal malnutrition (71), maternal stress (71), child abuse (72), and malnutrition (73, 74), are able to trigger these early neurodevelopment disruptions (65). A multi-genetic susceptibility for depression (75) can be modulated by one or more of these risk factors (76). Also, gene expression is susceptible of alterations by epigenetic mechanisms (77) such as DNA methylation (78–80). As consequence of these genetic dysfunctions both endocrinological alterations—e.g., HPA axis changes (81) and leptin alterations (65)—and immunological aberrations—e.g., elevated immune activity (82, 83)—are responsible for the disorder observed at the level of monoamines in depression—e.g., noradrenergic and serotonergic neurotransmission alterations (65, 84), being a BDNF modulation suggested as link between the endocrino-immunological alterations and the depressive phenotype (65).

In general, the neurodevelopmental theory of depression points out that very early and early stress factors in life are able to trigger the depressive phenotype later in life (65), which is accompanied by signs and symptoms, such as hippocampal (85) and amygdala atrophy (86), emotional and behavioral changes (87–90), and minor physical anomalies (Table 1) (91). Since microbiota has the potential to influence the brain during the development (92) and changes at the level of indigenous gut bacteria could be produced by stress (92), it has been noted that the MGB axis could be linked to the pathophysiology of depression.

### The Microbiome–Gut–Brain Axis and the Etiology of the Depressive Syndrome

The evidence presented above suggests that both the communication of the MGB axis and the bacterial modulation of the neurodevelopment involve three different systems that at the same time are strongly linked: the neurological system, the endocrine system, and the immune system. But, where could be the association between the MGB axis and the etiology of the depressive syndrome? There is, in the neurodevelopment! For example, very early exposition to valproic acid during the intrauterine life has been linked to both indigenous microbial alterations and behavioral changes later in life (93). Also, it has been demonstrated that early bacterial composition modulates neurodevelopment (94), including changes at the level of genetic expression of genes associated with brain function (94). This early neurodevelopmental modulation has the effect to influence adult behavior later in life (94). Moreover, it has been proposed, after hippocampal experimental results (95), that limbic system’s neurogenesis can be modulated by indigenous microbiota (95).

In the bidirectionality of the MGB axis, both gastrointestinal and neuropsychiatric pathological entities could be both origin and consequence of stress modulation (96). Stress factors during critical periods in the neurodevelopment—such as prenatal, postnatal/early childhood, and adolescence (65)—seem to be enough to develop the depressive phenotype (65). Since gastrointestinal bacteria have a strong communication with the brain *via* the MGB axis (96), gut microbiome alterations can be ideal etiological factors related to depression. As it is exposed above, it is not clear if there is a host–bacterial communication in the intrauterine life. If we consider the results published from a Spanish research group that suggest that the meconium from healthy neonates is not a sterile stool (53), being inhabit for bacteria such as *Enterococcus* and *Staphylococcus* (53), the GB communication could include the direct participation of bacteria, integrating a very early MGB axis in humans. Microglia are present early in the development (97). Microglia together with cytokines have a neuro-immune function in the early development (97, 98), being involved in the process of neuronal cytoarchitectural configuration (97, 98). If scientific results from studies *in vivo* suggest that very early immune activation has been linked with depressive symptoms later in life (99) and that gut bacteria can have an influence in early neurodevelopment *via* immune–endocrine communication (42), it could be possible to think that very early environmental stress factors associated with the etiology of depression modulate the risk to develop depression later in life *via* GB axis. Both microbiome alterations and MGB axis dysfunction have been linked to changes at the level of the HPA axis (100). Also, a normal HPA axis development requires an adequate gut indigenous bacteria composition early in life (43). The human development, including the neurodevelopment, is joined by systemic inflammation or inflamm-aging (Table 1) (101), which is part of a dysfunction of the immune system or immunosenescence (Table 1) (101). Apparently, there is an association between this systemic inflammation and the MGB axis (101), where stress is a risk factor for molecular, cellular, and behavioral alterations (101), including HPA axis dysfunction (101). Data from clinical reports suggest an association between the MGB axis and depression (102, 103), it has been reported that subjects with depression have elevated levels of IgA and IgM and the presence of gastrointestinal symptoms (102, 103). Animal studies have found that chronic gastrointestinal inflammation is linked to altered hippocampal neurogenesis (104). Also, a chronic elevated level of Il-6 was reported (104).

It has been established that a diet rich in fast-food, such as pizza, hamburgers, and donuts, has been connected to depression (105), increasing the risk for this syndrome (105). A diet, including fish and enough vegetables and fruits, can be a protective factor for depression (106), maybe because this kind of diet has an anti-oxidative and anti-inflammatory impact (106). Also, the inclusion of probiotics could have a positive effect for depressive patients (107), being suggested an anti-inflammatory and anti-oxidative effect induced by probiotics (107). Moreover, probiotics could have the potential to modulate growth factors (107), including the increment of BDNF levels (107).

## Conclusion

The evidence presented above suggests that the link between the MGB axis, the neuroendocrine–immune system—including energy homeostasis mechanisms—and the neurodevelopment is strong. Human neurodevelopment is a dynamic and long process that begins very early, during the intrauterine life, and continues during years, into the adulthood. These characteristics of the nervous system’s development—both its chronicity and dynamisms—make this process susceptible for alterations. Moreover, the origin of the GB axis takes place early during the prenatal period, directly after the beginning of the neurodevelopment. The GB bidirectional connection is present early in development, playing this network a key role modulating the formation of the brain. Numerous peripheral neurochemical signals together with intestinal microorganisms—which are host’s energy homeostasis elements—and a vagal communication are active regulator of both metabolism and immunity, mainly *via* the limbic system. Since the gastrointestinal tract has the first contact with diet elements—including the very early consumption of elements from mother’s vagina and excrement at birth—and diet is an important element defining human’s bacterial composition, then the interaction of host–bacteria *via* MGB axis is important regulating the neurodevelopment. The immune system regulates gastrointestinal bacterial composition. Very early nervous formation in the gastrointestinal tract is modulated for both immune cells and molecules. The origin of a depressive syndrome could be possible only in a disrupted brain. Apparently, the MGB axis plays an important role in the pathophysiology of depression. Gut microbiome modulation by environmental stress factors seems to contribute, together with genetic risk factors, to the development of this illness. Probably, these risk factors, which are potentialized by the combination of one or more other risk factors, induce a neuroendocrine–immune dysfunction that is the basis of the alterations observed in depressed subjects. The strong communication between the gastrointestinal system and the brain *via* the GB axis is present very early in the human development. This axis has a characteristic bidirectionality that seems to be attractive as antidepressant therapeutic target, being drugs that are able to induce changes at the inflammatory cascade proposed as antidepressants. Also, it has been recognized that gut microbiota play an important role in inflammation. Since gut bacteria have an important effect at the level of the GB axis and they are involved in both inflammatory and anti-inflammatory human functions, microbiota modulation by diet, probiotics, and drugs has been proposed recently as treatment strategy to improve the symptoms associated with the depressive syndrome. Antibiotics are environmental factors in the industrialized countries that induce important microbiota changes (108). However, interesting data have suggested that antibiotics such as minocycline have psycho-modulator effects (109), including antipsychotic and antidepressant functions (109). Apparently, these effects are associated with the anti-inflammatory, anti-oxidative, neuromodulator, and neuroprotective actions induced by minocycline (109). By an international systematic review and meta-analysis, it has been reported that anti-inflammatory drugs improve depressive symptoms (110). Interestingly, it has been reported that drugs, such as mood stabilizers, antipsychotics, and antidepressants, have anti-inflammatory effects (109). Moreover, an improvement of the pathways associated with the inflammatory cascades has been proposed as drug target in the treatment of depressed patients (111). Also, as results from different host–microbiota studies both innovative drug targets and new therapeutic strategies have been proposed with the objective of manipulate the human microbiome. These include: intestinal oxygen as therapeutic target (21), direct manipulation of human microbiota by fecal microbiota transplantation (21), alteration of microbiota products by diet (21), and improvement of drugs’ metabolism by microbiota’s modification (21).

Hippocrates of Kos, who lived around the fourth century BC (112), pointed out that diet plays an important role in the mental health (112). Hippocrates expressed this idea by his maxim: “Let food be thy medicine, and medicine be thy food” (112). Interestingly, this maxim is still being in our time of actuality. Individual lifestyles influence gut microbiota (5, 113), being our personal diet—e.g., a diet including food of plant and/or animal origin (113)—an important element to shape our own microbiome (5, 113). It has been noted that, during the extrauterine life, there are numerous transformations at the level of the gastrointestinal bacterial composition (96). Apparently, our diet has influenced the genome and the microbiome (5). Moreover, a better knowledge of our microbiome and how to modulate it could be helpful to improve existing medical strategies (9). Since the above evidence suggests that our early diet has a strong influence in our gut microbiome composition and in the susceptibility to develop different disorders, dietary practices such as breastfeeding during the first months of life, which improve our indigenous gastrointestinal bacteria and reduce the risk to develop future illness (114), should be intensely promoted. During the development, delivery mode plays an important role defining the characteristics of human microbiota (115, 116), being important differences in the acquired microbiota from vaginal delivered neonates compared to C-section delivered newborns (115, 116). Since early microbiota changes have been associated with the development of neurological alterations later in life, it could be possible that, in case of an elective delivery mode, a vaginal delivery should be strongly suggested to our patients, with the objective to reduce—since a neurobiological perspective—the risk for depression in the newborn. This is especially important if we consider that the delivery mode modulates gut microbiota composition particularly during the first 3 days of extrauterine life (116), an important period in human neurodevelopment.

A better perception of the significance of the MGB axis in the etiology of depression will help us to obtain a better understanding about the pathophysiology of this syndrome. Also, it opens the possibility to offer better treatment strategies to our patients. This will be possible only if we accomplish to decipher the genetic, anatomical, functional, and behavioral characteristics of the human microbiota. The most important limitation in the area of the MGB axis’ research lies in the fact that there is very little knowledge about the gut microbiome. There is necessary more scientific research that helps us to understand the relationship between the different microorganisms that inhabit the gastrointestinal tract. With the objective to understand the role played by microbiome in both human health and human disease, there are born different research consortiums around the world such as the “International Human Microbiome Consortium”^1^ (117), the Irish “Eldermet Project”^2^ (118), the German *Verbund OptiMD im Forschungsnetz für psychische Erkrankungen* or “OptiMD Consortium”^3^ (119), the international project “Metagenomics of the Human Intestinal Tract” or “MetaHIT Project”^4^ (120), and the American “NIH Human Microbiome Project”^5^ (121). In general, current scientific information is not able to explain the entire physiology of the MGB axis (122). Recently, it has increased the scientific research trying to elucidate the effects of gut microorganism in human behavior (122), including the link between both the vagal system and the neuroendocrine–immune system in brain function (122). There is still a wide scientific gap about the role played for the MGB axis in the etiology of different disorders, including neuropsychiatric illness. It is important to mention that the most of the scientific information about the MGB axis are product of animal research, being necessary the development of more human research to understand the host–microbiome interaction. However, it is evident that there is a strong host–microbiome connection, where environmental factors are important modulators of host’s gut microbiota. Apparently, our habits are able to modulate the MGB axis. Thus, an appropriate diet is of central importance for both a suitable neurodevelopment and an adequate mental health.

## Author Contributions

JL-O, RR, and TB contributed equally to this work.

## Conflict of Interest Statement

The authors report no biomedical financial interests or potential conflicts of interest. The reviewer, SB, and handling editor declared their shared affiliation and the handling editor states that the process nevertheless met the standards of a fair and objective review.

## Footnotes

^1^http://www.human-microbiome.org/.

^2^http://eldermet.ucc.ie/.

^3^http://www.optimd.de/.

^4^http://www.metahit.eu/.

^5^http://www.hmpdacc.org.
